# Development of a Novel Intervention (Mindful Steps) to Promote Long-Term Walking Behavior in Chronic Cardiopulmonary Disease: Protocol for a Randomized Controlled Trial

**DOI:** 10.2196/27826

**Published:** 2021-04-29

**Authors:** Daniel Litrownik, Elizabeth A Gilliam, Peter M Wayne, Caroline R Richardson, Reema Kadri, Pamela M Rist, Marilyn L Moy, Gloria Y Yeh

**Affiliations:** 1 Division of General Medicine Department of Medicine Beth Israel Deaconess Medical Center Boston, MA United States; 2 Osher Center for Integrative Medicine Harvard Medical School and Brigham and Women's Hospital Boston, MA United States; 3 Department of Family Medicine University of Michigan Ann Arbor, MI United States; 4 Pulmonary and Critical Care Section Department of Medicine Veterans Administration Boston Healthcare System Boston, MA United States

**Keywords:** mind–body exercise, internet-mediated intervention, behavior change, physical activity, COPD, heart failure

## Abstract

**Background:**

Despite current rehabilitation programs, long-term engagement in physical activity remains a significant challenge for patients with chronic obstructive pulmonary disease (COPD) and heart failure (HF). Novel strategies to promote physical activity in these populations are greatly needed. Emerging literature on the benefits of both mind–body interventions and web-based interventions provide the rationale for the development of the Mindful Steps intervention for increasing walking behavior.

**Objective:**

This study aims to develop a novel multimodal mind–body exercise intervention through adaptation of an existing web-based physical activity intervention and incorporation of mind–body exercise, and to pilot test the delivery of the new intervention, Mindful Steps, in a randomized controlled feasibility trial in older adults with COPD and/or HF.

**Methods:**

In phase 1, guided by a theoretical conceptual model and review of the literature on facilitators and barriers of physical activity in COPD and HF, we convened an expert panel of researchers, mind–body practitioners, and clinicians to inform development of the novel, multimodal intervention. In phase 2, we are conducting a pilot randomized controlled feasibility trial of the Mindful Steps intervention that includes in-person mind–body exercise classes, an educational website, online mind–body videos, and a pedometer with step-count feedback and goals to increase walking behavior in patients with COPD and/or HF. Outcomes include feasibility measures as well as patient-centered measures.

**Results:**

The study is currently ongoing. Phase 1 intervention development was completed in March 2019, and phase 2 data collection began in April 2019.

**Conclusions:**

Through the integration of components from a web-based physical activity intervention and mind–body exercise, we created a novel, multimodal program to impact long-term physical activity engagement for individuals with COPD and HF. This developmental work and pilot study will provide valuable information needed to design a future clinical trial assessing efficacy of this multimodal approach.

**Trial Registration:**

ClinicalTrials.gov NCT03003780; https://clinicaltrials.gov/ct2/show/NCT03003780

**International Registered Report Identifier (IRRID):**

DERR1-10.2196/27826

## Introduction

Physical activity is an important modifiable behavior that has enormous impacts on cardiopulmonary health. Chronic obstructive pulmonary disease (COPD) and heart failure (HF) are two systemic cardiopulmonary syndromes where patients experience similar morbidity and suffer debilitating decreases in physical activity. COPD, characterized by progressive airflow limitation most commonly due to environmental toxins (eg, smoking), confers considerable morbidity in up to 10% of US adults and is the third most common cause of death in the United States [1,2]. HF, also a progressive syndrome, is characterized by the inability of the heart to pump efficiently to meet metabolic demands and is associated with multiple cardiovascular and metabolic derangements [3,4]. Coexistence of the 2 conditions is being increasingly recognized, with estimated rates as high as 39% [5,6].

Patients with COPD and HF are characteristically deconditioned, with impairments in exercise tolerance and increases in dyspnea. Both self-reported and directly measured physical activity is significantly reduced, even at the earliest stages of disease [6-10]. During and following acute exacerbations, patients suffer further dyspnea, deconditioning, and reductions in physical activity [11-13]. Both cardiopulmonary conditions are also associated with multiple comorbidities, including anxiety, depression, and musculoskeletal disease, further contributing to reduced physical activity [6,14-16]. In HF, systematic reviews and meta-analyses report that exercise training reduces HF-related hospitalizations and results in clinically important improvements in health-related quality of life (HRQL) [17,18]. Walking, the most common form of exercise in these populations, has been associated with reduced HF risk [19]. The least active individuals with COPD have risks that are 2-6 times higher for acute exacerbations and hospitalizations than those most active [20]. Higher physical activity in COPD is also associated with a significantly lower hospital readmission rate and mortality risk, independent of lung function [21-23]. COPD studies examining daily step count support that every step walked can positively impact disease course [20,21,24,25].

Unfortunately, engagement in physical activity is a universal challenge amplified in these chronic cardiopulmonary populations. Conventional center-based cardiac and pulmonary rehabilitation have been shown to improve outcomes; however, they are vastly underutilized, not accessible to the population at large, and not sustainable [1-6,16,26-28] Providers refer less than 13% of potential candidates who would benefit from pulmonary or cardiac rehabilitation [29-32], and among those who are referred, noncompletion rates are as high as 20%-40% [31,33-36]. Despite improvements in exercise capacity, dyspnea, and HRQL after a typical course, benefits diminish 6-12 months after program completion [37]. Other outpatient and home-based programs have had variable success [38-40]. Qualitative studies have identified barriers and facilitators to long-term adherence to physical activity in COPD and HF. Important themes include addressing fears, promoting confidence, implementing personalized feedback and goals, providing peer support, and fostering a conducive environment with opportunities to engage in exercise [41,42]. Walking, in particular, has been identified as simple and accessible. Within this context, there has been interest in refocusing the current paradigm from one of promoting short-term aerobic exercise in structured settings to one of sustaining long-term everyday physical activity. Novel strategies to achieve this in these cardiopulmonary populations are greatly needed.

There is a burgeoning interest and emerging literature in mind–body interventions for fostering positive behavior change and physical activity [43-45]. In chronic cardiopulmonary disease, mind–body exercise, such as tai chi and yoga, may be particularly relevant [46,47]. Research has suggested that tai chi and mind–body breathing exercises are safe and feasible in older deconditioned patients and can lead to increases in HRQL, mood, exercise self-efficacy, and overall physical activity. In studies specific to COPD and HF, self-efficacy and overall empowerment have been shown to be important factors in facilitating long-term behavior change [40,48].

In addition, there is a rapidly emerging literature on use of web-based technology to promote healthy habits and behavior change [49-69]. Some home-based programs and lifestyle physical activity interventions, which combine supervised and independent exercise with self-monitoring devices, such as pedometers, have shown success in increasing physical activity [70-73]. One such web-based intervention, made specifically for elderly patients with COPD, increased HRQL and physical activity (daily step counts) in the short term (4-6 months) [74,75]. This multimodal intervention (Every Step Counts) created by Moy and Richardson includes an online interactive web platform coupled with a pedometer, step goal feedback, motivational and educational content addressing barriers to exercise, and an online community forum for social support. Development of this intervention was based on the behavioral theory of self-regulation, which emphasizes an iterative process of behavior change with individualized goal setting, iterative feedback, extrinsic motivating factors, and social support [76-78]. However, in long-term follow-up (12 months), benefits from this intervention were not sustained [79].

We hypothesized that by modifying this intervention with the addition and integration of mind–body principles, we could enhance existing key behavioral constructs and target new constructs through self-reflection, awareness, and personal transformation, which might lead to enhanced self-efficacy and the ultimate outcome of long-term adherence to walking behavior for COPD and HF. Thus, the current developmental study includes 2 phases with the following overarching aims: (1) adapt/refine the existing web-based physical activity intervention with added emphasis on mind–body principles and couple this platform with in-person mind–body exercise to create Mindful Steps; and (2) test delivery of Mindful Steps, a novel multimodal mind–body exercise intervention in a randomized feasibility trial with usual care control (N=42) in patients with COPD and/or HF.

## Methods

### Phase 1: Intervention Development

#### Conceptual Framework

**Figure 1:**
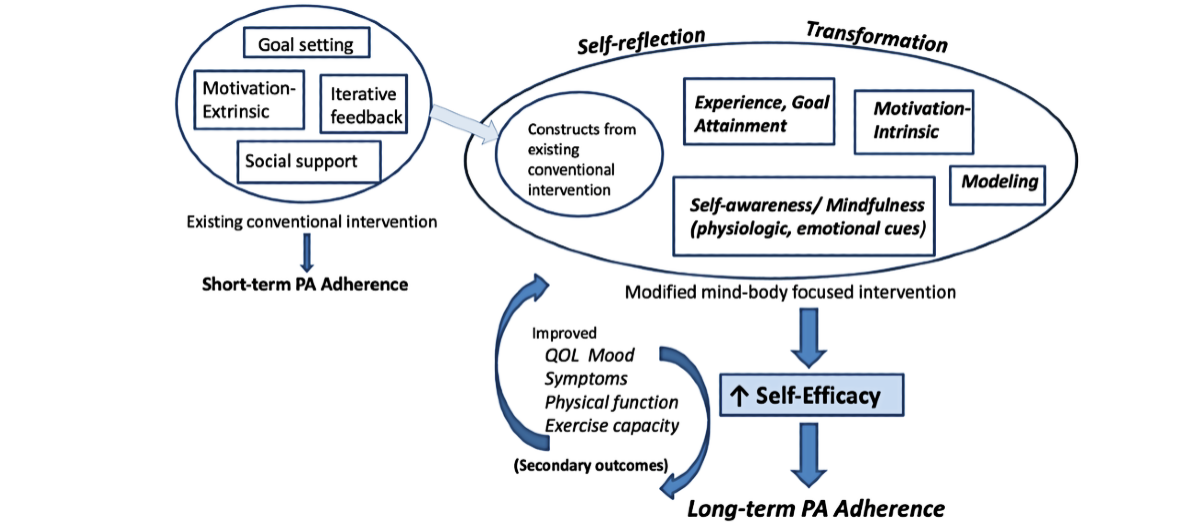
Conceptual model. PA; physical activity; QOL: quality of life.

Based on our experience with mind–body interventions and a review of the literature on facilitators and barriers of physical activity in COPD and HF, we developed an initial conceptual framework (Figure 1) that combined the existing Moy and Richardson intervention with elements of a mind–body exercise program to impact long-term behavior change.

We chose self-efficacy as a key outcome of interest. According to social cognitive theory, self-efficacy is one’s confidence in the ability to perform certain health behaviors and can influence the engagement in and actual performance of those behaviors, affecting health outcomes thereby [80]. Self-efficacy is a modifiable characteristic, and exercise self-efficacy is one of the strongest predictors of physical activity behavior [81]. Within our conceptual model, there are multiple characteristics of mind–body exercise that reinforce and enhance constructs of self-regulation from the existing intervention and additionally provide new constructs and potential mediating factors toward self-efficacy [82-84].

Experience/goal attainment, based on traditional self-efficacy theory [80], refers to the notion that prior successes boost confidence and facilitate self-efficacy. For example, mind–body exercise is often described as gentle, accessible, and nonthreatening, which may allow participants to achieve goals, even if in small increments. Similarly, small individual successes in iterative step-count goals further enhance that model with repeated success. *Modeling* describes when participants gain from seeing others succeed and modeling others’ behaviors. Prior qualitative data, including our studies of tai chi and HF, have reinforced the value of seeing others, perhaps even in a poorer condition than oneself, achieve success, which feeds confidence and motivation for one’s own abilities [82,83]. Self-awareness and mindfulness of physiologic cues and emotional states are inherently cultivated through mind–body exercise and are at the foundation of mind–body practices. Body signals such as fast heart rate or breathing due to anxiety or fear can negatively impact one’s perception of a particular task, such as exercise or walking. This may be illustrated by the anxiety–breathlessness cycle in COPD [85]. Strategies for mindfulness, body- and self-awareness, and concomitant acceptance and nonreactivity can mitigate this effect. Mind–body exercises can also address pain cycles and related exercise barriers [86-89] and impact fear of falling, which is correlated with self-efficacy and decreased falls [90,91]. *Intrinsic motivation* refers to motivation that is driven, not because of external factors (eg, a reward through an intervention website, feedback, or praise) but by internal factors, such as personal satisfaction and enjoyment. The inherent likeability of mind–body therapies is not well studied; however, numerous studies have reported qualitatively on enjoyment of mind–body exercise [82,84,88,92]. Certainly, increased mood with physical activity is well established. Finally, each of these potentially mediating factors are held within a larger domain of self-reflection and transformation that is a part of self-determination theory [93]. There is a growing literature that describes personal transformative experiences that can occur with mind–body practices [44–46].

In the modified Mindful Steps intervention, we retain the original constructs of goal setting, iterative feedback, extrinsic motivation, and social support that have appeared effective for short-term adherence and additionally introduce constructs that facilitate self-reflection and personal transformation through heightened, mindful, self-awareness and intrinsic motivation that can impact overall self-efficacy and facilitate long-term physical activity behavior [80,93,94].

#### Expert Panel

We assembled an expert panel to inform the creation and integration of a mind–body curriculum within the existing web-based platform. The panel included those with clinical and research expertise in mind–body exercise and tai chi, chronic cardiopulmonary disease (COPD and HF specialists), behavioral psychology, exercise physiology, and physical activity. We first began with open feedback from the core investigator team to develop the initial proposal for the curriculum. Using a modified Delphi process [95,96], we then solicited input from the larger expert panel on issues from practical implementation (eg, optimum ratio of and integration of in-person class contact with online video to decrease participant burden, modifications for safety, use of technology) to content-specific recommendations (eg, essential components of the mind–body curriculum, how best to integrate it with existing content, modifying disease-specific educational content for HF and already existing information for COPD). After a first round expert panel group meeting, individual interviews took place in an iterative fashion to gather further input and refine the protocol. After 2 subsequent group meetings with the expert panel, we arrived at a consensus protocol outlined in the following sections.

#### Mindful Steps: Intervention Components

The final Mindful Steps Intervention to be pilot-tested contains 7 primary components (Table 1). Each of the components is described below, with specific information on how it was modified from the prior Moy and Richardson intervention.

**Table 1:** Mindful Steps intervention components.

Intervention component	Description	Modifications from prior intervention	Rationale	Theoretical constructs
1. Pedometer–website interface with individualized step goal	Fitbit HR graphical display of daily/weekly steps Individualized daily step goals based on prior weekly step counts	No change	Allows accurate self-monitoring and personalized feedback of real-time progress Provides motivation to increase walking behavior Small, frequent successes in goal attainment leading to future successes	Goal setting Experience/goal attainment Extrinsic motivation Iterative feedback Self-efficacy
2. Motivational/educational website content	Motivational messages promoting walking and overcoming barriers Educational tips on disease self-management	Relevance of content expanded to HF population (in addition to COPD) Integrated mind–body content Expanded second 6 months with motivational memes and links to mind–body videos	Provides practical tools and strategies to promote walking, manage illness, and overcome barriers to walking	Extrinsic motivation Intrinsic motivation Self-efficacy Self-awareness and mindfulness
3. Online forum	Private online forum for study participants	Use of the forum by intervention instructors to facilitate engagement, sharing, and encourage walking	Provides community, social support, and sense of continuity with in-person classes	Social support Modeling Self-efficacy
4. Mindful walking curriculum videos	Weekly short educational video clips (2-8 minutes); theme-based (see section *Component 4: Mindful Walking Video Curriculum*) Short didactic teaching and guided mind–body exercises	New component	Provides practical tools and strategies for overcoming barriers to walking Cognitive reframing to find joy in walking Integrates mindfulness practices with everyday walking	Self-efficacy Intrinsic motivation Self-awareness and mindfulness
5. Mind–body exercise video library	Short videos (5-10 minutes) of mind–body exercises that support walking, including both standing and seated meditative exercises	New component	Reinforces in-person class learning Cultivates breath and body interoceptive awareness	Self-efficacy Self-awareness and mindfulness
6. Live group classes	In-person group class following Mindful Walking curriculum (see section *Component 4: Mindful Walking Video Curriculum*) Incorporating mindful walking, mind–body exercises, and facilitated group discussion	New component	Supports engagement with the website and step goal progress Allows time to unpack themes of weekly videos, practice and refine mind–body exercises Encourages social modeling of successes Provides community and social support	Self-efficacy Intrinsic motivation Self-awareness and mindfulness Modeling Experience/goal attainment Social support
7. Earn your stars	Online reward system that rewards reaching step goals, watching videos, and seeing motivational/ educational web content Small prizes for reaching 100 star and 500 star milestones	New component	Positive reinforcement of website engagement and walking behavior	Extrinsic motivation Self-efficacy

##### Component 1: Pedometer–Website Interface and Individualized Step-Count Goals

**Figure 2:**
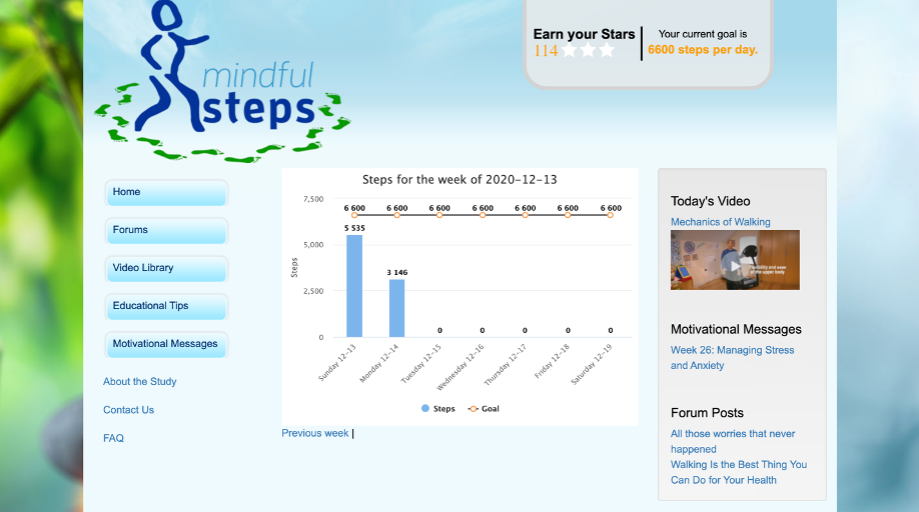
Mindful Steps home page.

The pedometer–website interface and individualized step-count goal components are unchanged from the original Moy and Richardson intervention [97]. Participants each receive a Fitbit Alta HR with a wristband and a personal account with access to the study web platform. An algorithm developed in prior studies is used to calculate individualized daily step-count goals each week, taking the average of the daily step counts from the prior week (most recent 7-day period of valid step-count data) and adding 400 steps [64,65,98,99]. Step-count goals do not exceed 10,000 steps per day. A graphical display of daily and weekly step counts and individual goals are highlighted on the home page allowing subjects to self-monitor progress in real time (Figure 2). Participants are asked to wear the pedometer at all times during waking hours and to sync the device daily for up-to-date feedback. Participants are encouraged to log into the website daily and also receive an automated weekly email with their new step-count goal for the coming week. Step-count goals provide extrinsic motivation to increase walking behavior over time.

##### Component 2: Motivational and Educational Website Content

The Motivational Messages and Educational Tips components are practical intervention tools and strategies to promote walking, manage symptoms of disease, and overcome barriers to physical activity. Motivational Messages (weekly message, total 52) includes titles such as “Incorporating Walking into your Daily Life,” “Managing Stress and Anxiety,” “Handling Set-Backs,” and “Keep Walking Fun.” Educational Tips (one tip every other day, total 88) consists of brief education information and includes titles such as “Use Your Shortness of Breath to Pace Yourself,” “Using Your Body Posture to Help You Feel Less Short of Breath,” “When Is Muscle Soreness Good?”, “Top 10 Reasons to Walk,” and “People Who Walk More Live Longer.” Educational Tips also includes links to external, publicly available websites that provide further material to explore the proposed topic (eg, the Heart Failure Society of America website information, “What if your Breathing Symptoms Worsen,” on exercise and symptom management for HF). Both Motivational Messages and Educational Tips were further adapted to include links to Mindful Walking curriculum videos (component 4) and mind–body exercise videos (component 5) as appropriate. Additionally, the Motivational Message component was modified to include prompts throughout for mindful awareness, brief guided mindfulness practices, emotion regulation techniques to address anxiety and fear, cognitive reframing of walking from a chore to a gift, and practical information on how and where to walk safely.

##### Component 3: Online Forum

A private forum was available on the web platform in the original Moy and Richardson intervention, but was underused [79]. In Mindful Steps, we aim to use the forum as an opportunity to engage participants in between live group classes (component 6). Prompts from class instructors and study staff are given to encourage dialogue and reflection on experiences with walking and further explore themes from classes, videos, Motivational Messages, and Educational Tips. The forum also provides an opportunity for participants to ask questions to the larger group, share successes and barriers to their daily walking and mindful practices, and offer helpful practical information, such as their favorite indoor walking spaces or outdoor trails. The purpose of the forum is to provide social support, social modeling of successes, and encourage community.

##### Component 4: Mindful Walking Video Curriculum

Based on feedback from our expert panel, we created brief (5-10 minute) videos designed to support walking as exercise, with each video centered around a particular theme (listed below).

***Theme***

1. Introduction: Body, Mind, and Breath
2. Mindful Warm-ups: Lower Body
3. Motivation to Move
4. Putting Joy Back into Exercise
5. Rewarding Yourself with the Gift of Walking
6. Mindfulness in Motion
7. Mindful Warm-ups: Upper Body
8. Renew Your Body with Your Breath
9. Every Step Counts
10. Self-Kindness
11. Overcoming Barriers and Challenges
12. Walking for Your Mind and Spirit
13. Pain Management
14. Preventing Falls
15. Strength and Flexibility
16. Breath Awareness
17. Your Body Affects Your Mind
18. Stop and Smell the Roses
19. Relaxation and Stress Management
20. Exploring Balance in Walking
21. Belly Breathing
22. Rest and Recharge along the Way
23. Importance of Posture
24. Leg Strength
25. Moderated Effort: the 70% Rule
26. Mechanics of Walking

A new video populates the Mindful Steps home page weekly (total 26; videos are repeated in the second 26 weeks). The video includes a short didactic teaching on the theme followed by theme-related guided mind–body exercises. For example, the Relaxation and Stress Management video includes a short teaching on the impact that mindful breath awareness can have on stress, mood, and symptoms of dyspnea, and is followed by a guided mindful breathing practice. The video concludes by encouraging viewers to practice mindful breathing while walking and to integrate these practices into activities of daily living.

The full video curriculum provides practical tools and strategies for overcoming barriers to walking by increasing awareness of cognitive, emotional, and bodily cues, and learning skillful ways to respond to these barriers, including pausing and slowing down, acceptance and nonreactivity, meditative breathing, active relaxation, and gentle stretches that alleviate common aches and pains. One recurring theme (also present in component 2) is the reframing of walking and exercise from being a chore or burden to being a gift, emphasizing the enjoyment and pleasure of walking, and thus cultivating a shift towards intrinsically motivated walking. The videos also support the application of mindfulness and mind–body techniques to walking itself as a way to support a more enjoyable, embodied, walking experience.

##### Component 5: Mind–Body Exercise Video Library

A library of 13 mind–body exercise videos used in our prior tai chi trials [100-103] are available on the website. These include the following: Swinging and Drumming, Swinging Up and Down, Hip and Leg Circles, Stretching Hands and Wrists, Spinal Cord Breathing, Song Breathing Shoulder Lifts, Dragon Wags Its Tail, The Fountain, Washing Your Body with Healing Energy, Renewing Your Body with Your Breath, Mindful Breathing, Belly Breathing, and Kindness Meditation. These videos reinforce the in-class learning of the mind–body exercises classes (component 6) and facilitate participants’ home practice of mind–body exercises. Collectively, the exercises include gentle aerobics, stretching, and meditative breathing, and aim to increase the range of motion and bring mindful awareness to the breath and individual parts of the body. They emphasize the importance of intention, facilitating ease and enjoyment of motion, and overall relaxation of body and mind.

##### Component 6: Live Group Classes

Live 75-minute group classes are offered to participants over the course of the year, with more instruction towards the beginning of the intervention period. Classes are conducted weekly for the first 6 weeks, biweekly for the next 34 weeks, and monthly for the last 12 weeks (25 total). Participants attend classes together as a cohort. The class curriculum follows themes from the Mindful Walking curriculum (component 4), including guided mindful walking, and also incorporates mind–body exercises from the video library (component 5). Classes begin and end with facilitated group discussion of class themes, exploration of barriers and successes with regard to step-count goals, and general check-ins. Classes are taught by experienced instructors who have led classes in our prior mind–body exercise clinical trials for over 10 years.

##### Component 7: Earn Your Stars

Earn Your Stars is an extrinsic motivational reward system to incentivize participants to stay engaged with the website content and their step-count goals. Participants can earn up to 3 stars a day for each of the following: reaching daily step-count goal, watching a video, clicking on Motivational Messages or Educational Tips. The star counter displays the total star accumulation on the individual’s home page. Small prizes (eg, US $5 gift cards) are given out when participants reach >100 and >500 stars.

### Phase 2: Pilot Randomized Controlled Trial

In a pilot randomized controlled trial, we are testing the delivery of the multimodal intervention versus usual care control in patients with COPD and/or HF. In addition to study and intervention feasibility and acceptability measures, we will explore the mediating components of the mind–body exercise approach articulated in our conceptual model and assess clinically meaningful changes in self-efficacy and overall physical activity over the long term of 1 year. At Beth Israel Deaconess Medical Center, 42 participants with COPD and/or HF have been randomized in a 2:1 ratio to participate in the 1-year Mindful Steps program or to receive usual care (including verbal and written instructions to exercise).

#### Study Population and Recruitment

The inclusion criteria include the following: age over >40 years; clinical diagnosis of COPD, defined as either a ratio of forced expiratory volume in one second (FEV_1_) to forced vital capacity <0.70 or chest computed tomography evidence of emphysema, and/or clinical diagnosis of HF syndrome (with left ventricular systolic dysfunction or preserved ejection fraction, and New York Heart Association Class 1-3); medical clearance from a provider to participate in an exercise program; an active email account with the ability to check email at least weekly; and access to a computer with an internet connection, USB port, and Windows. Meanwhile, the exclusion criteria include the following: self-reported COPD or HF exacerbation in the previous 2 weeks; inability to ambulate; clinical signs of unstable cardiovascular disease; hypoxemia during 6-minute walk test (oxygen saturation <85% with supplemental oxygen); inability to collect at least 7 of 14 days of baseline step counts; current participation in a cardiac or pulmonary rehabilitation program.

To identify potential participants, we searched hospital registries, screened physician schedules, solicited direct physician referrals through email, and used targeted patient advertising (paper flyers, digital screen ads, in-person presentations) in primary care or specialty clinics at Beth Israel Deaconess Medical Center and pulmonary rehabilitation clinics in the community. This was followed by opt-in mailing and telephone outreach conducted by our research assistants.

#### Randomization and Intervention Delivery

As in prior studies, we used a 14-day run-in period of pedometer (Fitbit) use requiring at least 7 days of valid data (>200 steps per day) as a criterion for participants to be eligible for randomization. As the sample size is relatively small, we will use blocked randomization with varying block sizes and a computer-generated sequence of random numbers with a concealed and unpredictable allocation scheme.

Participants in the Mindful Steps intervention will receive the pedometer, access to the Mindful Steps website, and a schedule of in-person mind–body exercise classes as described in detail previously in this paper. All participants will continue pharmacological treatment and receive care through their usual providers. Participants in both groups will also receive an education handout from Harvard Health Publishing and Harvard Medical School titled “Walking for Health,” which includes information on the health benefits of walking, how to get started on a walking program, and specific walking workouts [104].

#### Study Assessments

Table 2 outlines study feasibility and intervention acceptability and adherence measures that are being tracked to inform a future larger trial.

**Table 2:** Study feasibility and intervention acceptability and adherence measures.

Outcomes and measures		Description
**Study feasibility**		
	Recruitment	Track recruitment rate by site and strategy (eg, clinic visits, mailings, advertisement sites, involvement of physician, etc.)
	Retention	Track retention rate with acceptable retention defined as <20% dropouts
**Intervention acceptability**		
	Qualitative interview	Semistructured interview at 6 months (in person) and 12 months (by phone) to assess patient experience, understand helpful components of the multimodal intervention, and specifically explore themes related to conceptual model
	Physical Activity Enjoyment Scale [105]	8-item shortened version used to assess enjoyment of physical activity
**Intervention adherence**		
	MBE^a^ adherence	Track MBE class attendance, online video tutorial use, and logs of home practice
	Web platform usage	Track logins to the website, clicks on education links, downloads and views of videos, and pedometer use

^a^MBE: mind–body exercise.

In addition, all participants will undergo testing to collect exploratory outcome measures at baseline, month 3 and 6 in-person, and month 9 and month 12 by phone. Table 3 outlines exploratory patient-centered outcomes including cognitive-behavioral and psychosocial measures, overall physical activity, disease-specific HRQL and symptoms, and physical function and exercise capacity.

At each time point, we will administer questionnaires and collect physical activity data. At in-person visits only, we will additionally perform the 6-minute walk test. All tests will be conducted by study staff who will be blinded to treatment assignment. We will also track potential adverse events and COPD- and HF-related exacerbations and hospitalizations at each testing visit to assess safety.

Qualitative interviews at 6 months and 12 months will further assess facilitators and barriers to participation and acceptability of the intervention and its multiple components. Additional questions will be guided by our conceptual model and explore themes of motivation, goal attainment, social support, self-reflection, and personal transformation.

**Table 3:** Patient-centered outcomes.

	Measure	Description
**Cognitive–behavioral and psychosocial**		
	Self-Efficacy for Exercise Scale [106]	9-item validated scale from McAuley’s original barriers scale
	Self-Efficacy for Managing Chronic Disease Scale [107]	6-item subscale from the Chronic Disease Self-Management Study
	Intrinsic Motivation Inventory [108]	22-item validated scale with 4 subscales: interest/enjoyment, perceived competence, perceived choice, and pressure/tension
	Patient Activation Measure [109]	13-item short form validated scale from Insignia Health
	Medical Outcomes Study Social Support Survey [110]	20-item validated scale with 5 subdomains of tangible support, emotional support, informational support, affectionate support, and positive social interaction
	Multidimensional Assessment of Interoceptive Awareness [111]	32-item validated scale with 5 subscales of body sensations, emotional reaction/attentional response, attention regulation, mind–body integration, trusting body sensations, assessing 8 domains of body awareness
	CES-D^a^ [112]	20-item validated scale; participants with CES-D score > 20 will be referred back to primary provider for evaluation
**Physical activity**		
	Community Healthy Activities Model Program for Seniors Physical Activity Questionnaire [113]	41-item validated scale with leisure, household, occupational physical activity domains; allows estimation of weekly caloric expenditure
	Pedometer [114]	Step counts measured by Fitbit pedometer; device worn during waking hours except when showering/bathing
**Disease-specific HRQL^b^**		
	St. George’s Respiratory Questionnaire [115,116]	Respiratory-specific extensively validated measure of HRQL; total score and subscales of activity, symptoms, and impact
	Minnesota Living with HF^c^ [117]	HF disease–specific extensively validated measure of HRQL
	mMRC^d^ Dyspnea scale [118]	5-item scale assessing breathlessness (part of BODE^e^ index)
**Physical function and exercise capacity**		
	PROMIS^f^ Physical Function, Fatigue [119]	Short form 7-10 items; from NIH^g^ toolbox PROMIS
	6-minute walk test [120]	Distance walked in 6 minutes as a measure of exercise capacity, performed according to ATS^h^ guidelines; subjects will use supplemental oxygen if already prescribed oxygen during activity

^a^CES-D: Center for Epidemiological Studies Depression scale.

^b^HRQL: health-related quality of life.

^c^HF: heart failure.

^d^mMRC: Modified Medical Research Council.

^e^BODE: body-mass index, airflow obstruction, dyspnea, and exercise.

^f^Patient-Reported Outcomes Measurement Information System.

^g^NIH: National Institutes of Health.

^h^ATS: American Thoracic Society.

#### Statistical Analysis

For study feasibility and intervention adherence, we will use descriptive statistics to evaluate recruitment and retention rates, attendance at mind–body exercise classes, and adherence with the online intervention (web-platform usage). We will consider study retention successful if the retention rate is at least 80% and if attendance at in-person classes is at least 70% (17/25 classes). Mean scores on the Physical Activity Enjoyment Scale at each time point will be reported [105].

To further evaluate intervention acceptability, 6-month and 12-month qualitative interviews will be recorded and transcribed verbatim. Thematic analysis will be informed by grounded theory methods based on our semistructured questions. Transcripts will be independently coded by at least 2 separate investigators to identify emergent themes in an iterative process until thematic saturation is reached. We will particularly search for themes that may give insights into factors mediating a shift in self-efficacy towards longer-term maintenance of walking and overall physical activity.

For each patient-centered outcome, repeated measures analyses will be performed using SAS PROC MIXED to evaluate trajectories over the course of the 4 time points for each group and the differences between the groups in these trajectories at each time point. Planned paired comparisons and Cohen’s d effect sizes will also be computed to evaluate the magnitude of improvement between time points and group differences in change. These data will provide valuable information including what outcome domains and measures may demonstrate clinically meaningful changes in future models, the appropriate number of time points to use in a future clinical trial, and measures of variability within and between groups to inform the design of future studies. Measures considered sensitive to change will be those that indicate trends in improvement over time.

## Results

Mindful Steps was funded in February 2017, approved by the institutional review board at Beth Israel Deaconess Medical Center in January 2017 and at the University of Michigan in May 2017. Phase I intervention development was completed in March 2019, phase II data collection began in April 2019, and completion is expected by August 2021. A total of 41 subjects have been enrolled.

## Discussion

Through the integration of a web-based physical activity intervention with a mind–body video curriculum and in-person mind–body exercise, we created a novel, multimodal program with the goal of increasing long-term physical activity adherence for patients with COPD and HF. Guided by a conceptual model, the intervention aims to target multiple factors underlying behavior change that collectively act to enhance and internalize self-efficacy, which may lead to a longer-term, more sustainable shift in physical activity behavior. This pilot will provide valuable information needed to design a future clinical trial further assessing efficacy of this multimodal approach.

## Appendix

Multimedia Appendix 1 Peer-review report.

## Abbreviations

COPD: chronic obstructive pulmonary disease
HRQL: health-related quality of life
HF: heart failure
