# Supplementary material for: Development of a Novel Intervention (Mindful Steps) to Promote Long-Term Walking Behavior in Chronic Cardiopulmonary Disease: Protocol for a Randomized Controlled Trial
Source: JMIR Res Protoc. 2021 Apr 29;10(4):e27826. doi: 10.2196/27826 (PMC8120426; doi:10.2196/27826)
Supplement: Multimedia Appendix 1 [file resprot_v10i4e27826_app1.pdf]

1R34AT009354-01 Yeh, Gloria

RESUME AND SUMMARY OF DISCUSSION: This application proposes to develop a multimodal physical activity intervention for chronic obstructive pulmonary disease (COPD) and chronic heart failure (HF) older adults by adapting an existing web-based intervention incorporating mind-body principles and mind body exercises. As the numbers of COPD and HF individuals, and the social burden are high, success of this pilot study could have significant impact. The strong multidisciplinary investigative team has impressive and relevant set of preliminary studies to support current application. Innovative web-based mindfulness intervention combined with mindful breathing and physical activity can potentially address medical needs of home-bound and rural patients. Conceptual model for rationale of the study and outcomes is excellent. Proposed two-phase design of development then testing will more likely lead to successful implementation. Combining two similar chronic illnesses lends itself to generalizability and could impact a larger population. Research environment is outstanding and highly qualified to carry out proposed study. There are some concerns over minor weaknesses. Utilization of a standard care control is not well justified and the large measurement battery could represent considerable participant burden. However, the weaknesses are very minor and strengths of the application far outweigh the weaknesses.

DESCRIPTION (provided by applicant): Physical activity is an important modifiable behavior that has enormous impacts on chronic cardiopulmonary health. Chronic obstructive pulmonary disease (COPD) and chronic heart failure (HF) are similar systemic syndromes. Both populations are often are deconditioned, suffer exercise intolerance and dyspnea, and have co-morbidities such as anxiety, depression, and musculoskeletal limitations. Both are notoriously difficult to engage in and sustain physical activity. While exercise improves outcomes, current programs are often underutilized and inaccessible, and long term adherence is problematic. There is a robust literature on the benefits of various mind-body exercises for multiple conditions,. Specifically in our studies of tai chi and mind-body breathing for HF and current investigations in COPD, we have shown improvements in quality of life, exercise capacity, mood, physical activity, with data suggesting that self-efficacy may be an important factor to facilitate long term behavioral change. There is also a growing body of literature examining use of emerging web-based technology to promote healthy habits. While there is interest in intervention delivery modalities that are home-based or use web-based forums, this area of mind- body exercise is much less studied. We have previously developed a conventional web-based physical activity intervention which includes an online interactive platform, providing physical activity feedback from a pedometer, individualized graphs of progress, motivational and educational content, and a community forum for social support. While this intervention increases physical activity in the short term, we have seen that in patients with COPD, this benefit wanes by12 months. Further novel strategies to address long term adherence to physical activity in both COPD and HF are still needed. We plan to address this gap by leveraging our experience in both mind-body therapies and web-based physical activity intervention delivery. In this proposal, we will adapt the existing conventional web-based intervention to incorporate mind- body principles and mind-body exercise, to develop a novel, multimodal physical activity intervention for COPD and HF ("Mindful Steps"). Through the integration of mind-body exercise, this interactive online platform will support mind-body practice and promote the incorporation of mindfulness into daily activities (e.g., mindful walking.) Based on established behavioral theories, we propose that the enhanced intervention may better facilitate overall and exercise-related self-efficacy. Our overarching goal will be test this intervention in a future randomized clinical trial (as compared to the existing conventional intervention and to usual care) to increase self-efficacy and long-term physical activity adherence. The aims of this R34 grant are 1) to adapt the existing web-based physical activity intervention with an emphasis on mind-body principles and couple this platform with in-person mind-body exercise training and 2) to test delivery of this multimodal intervention ("Mindful Steps") in a randomized feasibility trial (N=42 total) with usual care control in patients with COPD and HF.

PUBLIC HEALTH RELEVANCE

Physical activity is an important modifiable behavior that has enormous impacts on the health of persons with chronic lung and heart disease. An interactive, educational and motivational online platform which emphasizes mindful movement, together with in-person mind-body exercise classes, may be helpful to increase confidence to exercise and promote long-term physical activity.

CRITIQUE 1:

Significance: 2
Investigator(s): 1
Innovation: 2
Approach: 4
Environment: 1

Overall Impact:

This study, proposed by experienced investigators, seeks to adapt an existing web-basee physical activity intervention to include mind-body principles for patients with COPD and heart failure (HF). This R34 grant aims to adapt the interventions into a web-based protocol and then to pilot test the intervention on 42 patients with COPD or HF. This intervention is novel and capitalizes on Dr. Yeh and Moy's previous research in web-based walking programs and Tai Chi. It is unclear exactly how these interventions will be adapted for a web-based program but if they are able to accomplish this, it would be highly impactful. The study is rigorously designed using tools that are both reliable and valid. The pilot data will provide needed data for an eventual R01 application.

1. Significance:

Strengths

- Longterm sustainability of physical activity for patients with both COPD and HF is difficult
- Patients with COPD and HF are inactive and would benefit from such interventions

Weaknesses

- It's unlear what the added benefit of "mindful steps" over the current web-based programs would be

2. Investigator(s):

Strengths

- This is a very strong investigative team.

Weaknesses

- No concerns

3. Innovation:

Strengths

- Development of a mindfulness intervention combined with mindful breathing and physicial activity is innovative
- Putting this intervention on a web-based platform is innovative

Weaknesses

- None noted

4. Approach:
Strengths

- Thoughtful consideration of the development of the mindful steps intervention
- RCT feasibility / pilot study
- Well-planned analytic plan
- Intervention can by far-reaching since it is web-based
- Existing platform on which to build the intervention

Weaknesses

- It's unclear how the mindfulness component will be delivered via the web

5. Environment:
Strengths

- Strong environment

Weaknesses

- No concerns

Protections for Human Subjects:
Acceptable Risks and/or Adequate Protections

- This is a low risk study

Data and Safety Monitoring Plan (Applicable for Clinical Trials Only):
Acceptable
  - A monitoring system is in place

Inclusion of Women, Minorities and Children:

- Sex/Gender: Distribution justified scientifically
- Race/Ethnicity: Distribution justified scientifically
- Inclusion/Exclusion of Children under 18: Excluding ages <18; justified scientifically
- HF and COPD generally occurs in older adults

Vertebrate Animals:
Not Applicable (No Vertebrate Animals)

Biohazards:
Not Applicable (No Biohazards)

Authentication of Key Biological and/or Chemical Resources:

Not Applicable (No Relevant Resources)

Budget and Period of Support:

Recommend as Requested

CRITIQUE 2:

Significance: 2
Investigator(s): 2
Innovation: 1
Approach: 4
Environment: 1

Overall Impact:

This project proposes to adapt an existing conventional web-based intervention by incorporating mind-body principles and Mind Body Exercises (MBEs) to develop a multimodal physical activity (PA) intervention for COPD and HF ("Mindful Steps") in older adults. This proposal was well written and organized. Study builds upon prior research with a well-developed PA web-based intervention that showed short-term benefit but waned by 12 months. Other research by the investigators showed that an in-person MBE (i.e. Tai Chi) intervention was successful at not only improving measures of PA but also important psychological variables such as self-efficacy and empowerment. This project will attempt to merge the two interventions together to deliver both content through an on-line and in person multimodal delivery system. Strengths of the project include a strong investigative team with relevant expertise and experience working together. Builds upon past prior research in relevant areas. Significance of the project is high since updating delivery models utilizing technology to decrease participant burden while simultaneously attempting to influence psychological health through the incorporation of MBE's could provide important information and infrastructure to further advance the field. In addition, the targeting of two similar chronic illnesses lends itself to generalizability and could impact a larger number of individuals. The incorporation of both traditional PA exercises along with more alternative MBE exercises lends itself to a novel integrated approach. Weaknesses include no junior faculty on team. Justification for utilizing a standard care control and for not utilizing power estimates is not considered scientifically sufficient. Measurement battery is large and could represent considerable participant burden. No information is given on how participants will be compensated for time and effort for assessments and whether procedures are in place to help older participants who could potentially have cognitive difficulties given the older age of the population to be recruited.

1. **Significance:**

Strengths

- PA is an important modifiable behavior that has enormous impacts on the prognosis of Chronic obstructive pulmonary disease (COPD) and chronic heart failure (HF) and adherence to PA recommendations is frequently problematic.
- Psychosocial determinants such as depression, low self-efficacy, and low social support also contribute to low levels of PA in high-risk populations.
- Prior data from the investigative team shows that MBEs, though of low PA intensity, still have the power to significantly influence health and psychological outcomes.

- Adapting an intervention for two similar chronic illnesses lends itself to generalizability.
- Updating delivery models utilizing technology to decrease participant burden while simultaneously attempting to influence psychological health through the incorporation of MBE's could provide important knowledge and infrastructure to further advance the field.
- This stage of treatment development is justified before moving on to a larger randomized trial.

Weaknesses

- None noted

2. Investigator(s):

Strengths

- Investigative team is excellent and has considerable experience in the population of interest.
- A team of well-known consultants with expertise in mind body principles and MBE interventions lend itself to project success.
- Team has extensive history of NIH funded projects.

Weaknesses

- Investigative team appears to be top heavy with more seasoned faculty. The inclusion of junior investigators would make the team more well-rounded.

3. Innovation:

Strengths

- Investigating new delivery systems for MBE (i.e. home-based or web-based forums) in high risk populations has not been extensively studied.
- The incorporation of both traditional PA exercises along with more alternative MBE exercises lends itself to a novel integrated approach.

Weaknesses

- None noted

4. Approach:
Strengths

- Based on formative pilot work highly relevant to the proposed project.
- Adaptation to come from existing treatments with prior research.
- Use of a multi-modal delivery system that still allows for some in-person contact.

- Inclusion of both PA and important psychosocial determinants of health.
- Benchmarks for success including acceptability and adherence are well thought out throughout design.
- The inclusion of a commercially-available pedometer (FitBit) to increase motivation as well as measurement.

Weaknesses

- The use of a standard care control weakens the interpretation of findings and is not well justified. Though the investigators describe alternative strategies they considered in regards to groups, stating that they chose the design based on limited resources is not scientifically sufficient.
- Though it is understood that treatment development projects typically under powered, effects sizes can be used as a benchmark of significance. No consideration to this issue was addressed and stating that the proposed sample size was based on time and costs is again not scientifically sufficient justification.
- The investigators state that the primary variables of interest include self-efficacy and overall physical activity level. However, the assessment battery consists of many other variables that seem to represent considerable burden to the participants.
- No information is given on how participants will be compensated for time and effort for assessments and whether procedures are in place to help older participants who could potentially have cognitive difficulties given the older age of the population to be recruited.
- No details are given as to whether FitBit sensors are able to effectively measure PA without bias or error.

5. Environment:

Strengths

- The institutions are ideal for the proposed work.
- VA Boston Healthcare System appears to have sufficient recruitment resources.

Weaknesses

- None noted

Protections for Human Subjects:

Unacceptable Risks and/or Inadequate Protections

- No information is given on how participants will be compensated for time and effort for assessments and whether procedures are in place to help older participants who could potentially have cognitive difficulties given the older age of the population to be recruited.

Inclusion of Women, Minorities and Children:

- Sex/Gender: Distribution justified scientifically

- Race/Ethnicity: Distribution justified scientifically
- Inclusion/Exclusion of Children under 18: Excluding ages <18; justified scientifically

Vertebrate Animals:

Not Applicable (No Vertebrate Animals)

Biohazards:
Not Applicable (No Biohazards)

Authentication of Key Biological and/or Chemical Resources:
Not Applicable (No Relevant Resources)

Budget and Period of Support:
Recommended budget modifications or possible overlap identified:

- Project Coordinator and Research Assistant FTE seem a little low for project responsibilities.

CRITIQUE 3:

Significance: 2
Investigator(s): 1
Innovation: 3
Approach: 2
Environment: 1

Overall Impact:
This application aims to adapt an existing web-based intervention to incorporate mind-body practices to adults with COPD and HF. Given the large burden of these diseases, and lack of behavioral adherence to physical exercise, could have an major impact if mind-body practices improve upon the present intervention. The conceptual model presented for the rationale of the study and outcomes is excellent. The investigators are well poised to execute the research project based on prior work. Generally, the approach is rigorously designed as it relates to study design, recruitment, outcome selection, and proposed analyses. The comparison group does not advance or prepare the investigators for the next larger study. A comparison group that also uses an online platform and/or groups with time and attention matched would be more informative. This would be especially valuable given that the intervention under investigation is multimodal with possibly many active components/factors.

1. Significance:

Strengths

- Cardiopulmonary diseases produce significant morbidity with improvement in physical activity, and while exercise is helpful, adherence is poor. Improving adherence through technology and/or mind-body practices may be very beneficial.
- The study team has demonstrated use of web-based physical activity intervention and separately mind-body practices for this population. The objective of the grant is to deliver mind-body practices on this platform, which is a logical collaborative project.
- The theoretical framework presented as the rationale and conceptual model for the proposal is excellent and well presented.

Weaknesses

- It is a very practical approach to combine CHF and COPD for this grant and it is appreciated that a significant proportion of the population have both conditions. However, it is not clear if anything is lost by combining the two with regards to a targeted intervention, and/or measurement of outcomes.

2. Investigator(s):

Strengths

- The investigators have substantial experience to conduct mind-body clinical trials, behavioral measurements, and develop and assess delivery of the intervention on the web-based platform.

Weaknesses

- None noted.

3. Innovation:

Strengths

- Online mind-body content for these specific populations has not been developed and rigorously evaluated.
- Supplementing usual care with mind-body practices to boost long-term adherence is novel

Weaknesses

- Outcomes selected are not particularly novel

4. Approach:

Strengths

- Study design is organized in two phases of development and then testing which is appropriate and will more likely lead to successful implementation
- Building mind-body practices into an existing intervention that is successful is practical and can evaluate potential value added from mind-body practices
- Frequency of classes in-person versus online videos is part of research question for first phase and will allow for decision as to what dose and method of intervention delivery is appropriate.

Weaknesses

- The intervention is multi-modal with various components which will make it difficult to determine which components were most helpful or useful
- Investigators discuss that they considered other study designs for phase 2 including mind-body intervention versus conventional intervention versus usual care, but opted the present choice to see if patients were willing the be randomized. Given the substantial experience and prior studies of this group, they have demonstrated feasibility of randomization in other clinical trials. It is not clear why the present pilot would be different. Selection of a usual care group consisting of just educational materials will not provide substantial useful information. A more comparable intervention, preferably with time and attention matched and even method of delivery would be more informative. For example, in-person educational groups along with supplemented online content (such as the conventional intervention or even watered down) would be more informative. Also, a comparison group could have been selected to specifically prepare to compare an outcome of major interest in a future clinical trial; such as online engagement long-term or self-efficacy by the specific addition of mind-body practices to the existing conventional intervention.

5. Environment:

Strengths

- The clinical centers and investigators are well-equipped to recruit this clinical population and implement the intervention

Weaknesses

- None noted.

Protections for Human Subjects:

Acceptable Risks and/or Adequate Protections

Data and Safety Monitoring Plan (Applicable for Clinical Trials Only):

Acceptable

Inclusion of Women, Minorities and Children:

- Sex/Gender: Distribution justified scientifically
- Race/Ethnicity: Distribution justified scientifically
- Inclusion/Exclusion of Children under 18: Excluding ages <18; justified scientifically

Vertebrate Animals:

Not Applicable (No Vertebrate Animals)

Biohazards:

Not Applicable (No Biohazards)

Authentication of Key Biological and/or Chemical Resources:

Not Applicable (No Relevant Resources)

Budget and Period of Support:

Recommend as Requested

CRITIQUE 4:

Significance: 2
Investigator(s): 2
Innovation: 5
Approach: 9
Environment: 1

Overall Impact:

The aims of the proposed study are to: 1) adapt/refine existing web-based PA intervention with added emphasis on mind-body principals, and to couple this platform with in-person MBE training; 2) test the delivery of ‘Mindful Steps’ in a randomized feasibility trial with usual care control in patients with COPD and HF (provided by applicant). The proposed study contains a number of strengths. The multi-disciplinary investigative team is very strong, and they collectively have an impressive and relevant set of preliminary studies. The populations (COPD and HF) are in need of effective treatments with sufficient uptake. The number of individuals experiencing COPD and HF are high, and the societal burden is high. The adaptation process is clearly delineated and well-informed, and the multi-modal intervention is creative and could result in a high effective intervention. The theoretical is grounded in existing research and effectively integrates the PA and mindfulness literatures. The study also has a number of weaknesses which limits its contribution to the field. A significant weakness in the inclusion of in-person meetings, which obviates the ability of the intervention to increase service access to homebound or rural patients. Other weaknesses include the lack of information regarding the ecological momentary assessment approach, the vagueness regarding self-reflection and personal transformation, and not identifying members of the consensus panel.

1. Significance:

Strengths

- The proposed study examines two conditions that affect a sizeable number of people
- The proposal identifies the limitations of existing physical activity (PA) interventions
- The theoretical model is well-integrated across the PA and mindfulness literatures

Weaknesses

- There is not an evidence base for the role of intrinsic motivation in MBEs
- Self-reflection and transformation are not well operationalized; the text in the proposal on these factors appear related to the self-efficacy construct, which is a core component of the theoretical model

2. Investigator(s):

Strengths

- The proposal includes an inter-disciplinary team
- The investigators are highly skilled and bring a depth of experience

Weaknesses

- The team is large, and may contain some duplication

3. Innovation:

Strengths

- The inclusion of web-based technology is an innovative approach and has the potential to address medical needs of home-bound and rural patients

Weaknesses

- I do not consider ‘the unique qualities of mind-body approaches’ to be innovative.

4. Approach:

Strengths

- The adaptation process is clearly delineated, and provides relevant information regarding the key domains that will inform the adaptation.
- The multi-modal intervention builds on strengths of two bodies of literature and has the potential to result in a creative, research-informed intervention.

Weaknesses

- The biweekly in-person groups obviate the ability to accommodate rural or homebound patients, which is a key strategy to increase service access. Support could be given via an online support group.
- It would have been useful to already have identified and have support from experts in the field who would serve on the consensus panel.
- More information is needed on the online assessments using an ecological momentary assessment approach.

5. Environment:

Strengths

- The institutions are highly qualified to carry out the proposed study.

Weaknesses

- None

Protections for Human Subjects:
Acceptable Risks and/or Adequate Protections

Inclusion of Women, Minorities and Children:
- Sex/Gender: Distribution justified scientifically
- Race/Ethnicity: Distribution justified scientifically
- Inclusion/Exclusion of Children under 18: Excluding ages <18; justified scientifically

Vertebrate Animals:
Not Applicable (No Vertebrate Animals)

Biohazards:
Not Applicable (No Biohazards)

Authentication of Key Biological and/or Chemical Resources:
Not Applicable (No Relevant Resources)

Budget and Period of Support:
Recommend as Requested

THE FOLLOWING SECTIONS WERE PREPARED BY THE SCIENTIFIC REVIEW OFFICER TO SUMMARIZE THE OUTCOME OF DISCUSSIONS OF THE REVIEW COMMITTEE, OR REVIEWERS' WRITTEN CRITIQUES, ON THE FOLLOWING ISSUES:

PROTECTION OF HUMAN SUBJECTS (Resume): ACCEPTABLE
However, no information is given on how participants will be compensated for time and effort for assessments and whether procedures are in place to help older participants who could potentially have cognitive difficulties given the older age of the population to be recruited.

INCLUSION OF WOMEN PLAN (Resume): ACCEPTABLE

INCLUSION OF MINORITIES PLAN (Resume): ACCEPTABLE

INCLUSION OF CHILDREN PLAN (Resume): ACCEPTABLE

COMMITTEE BUDGET RECOMMENDATIONS: The budget was recommended as requested.

Footnotes for 1 R34 AT009354-01; PI Name: Yeh, Gloria Y
